# Effectiveness of Interventions for Reducing Loneliness Among Adult Cancer Survivors: A Systematic Review and Meta-Analysis

**DOI:** 10.3390/cancers18193101

**Published:** 2026-09-24

**Authors:** Celia Sánchez-Gómez, Alba Sánchez-Gil, Eduardo José Fernández-Rodríguez, Jesus Perez, Emilio Fonseca-Sánchez, Sara Jiménez-García-Tizón, Alberto García-Martín, Javier Martín-Vallejo, Juan Luis Sánchez-González

**Affiliations:** 1 Department of Developmental and Educational Psychology, Faculty of Psychology, University of Salamanca, Av. de La Merced, 109, 37005 Salamanca, Spain; celiasng@usal.es; 2 Institute of Biomedical Research of Salamanca (IBSAL), 37007 Salamanca, Spain; edujfr@usal.es (E.J.F.-R.); jesusperez@usal.es (J.P.); efonseca@usal.es (E.F.-S.); albergm@usal.es (A.G.-M.); jmv@usal.es (J.M.-V.); juanluissanchez@usal.es (J.L.S.-G.); 3 National Centre of Reference for People with Alzheimer’s Disease and Other Dementias, 37008 Salamanca, Spain; albasanchezgil.sg@gmail.com; 4 Department of Nursing and Physiotherapy, University of Salamanca, 37007 Salamanca, Spain; 5 Faculty of Medicine, Department of Psychiatry, University of Salamanca, 37007 Salamanca, Spain; 6 Department of Psychiatry, University of Cambridge, Cambridge CB2 0SZ, UK; 7 Cambridgeshire and Peterborough NHS Foundation Trust, Cambridge CB21 5EF, UK; 8 Norwich Medical School, University of East Anglia, Norwich NR4 7TJ, UK; 9 Department of Medical Oncology, University Hospital of Salamanca, 37007 Salamanca, Spain; 10 Department of Labor Law and Social Work, Faculty of Law, University of Salamanca, 37007 Salamanca, Spain; 11 Faculty of Medicine, Department of Statistics, University of Salamanca, 37007 Salamanca, Spain

**Keywords:** cancer, loneliness, meta-analysis, psychosocial interventions, systematic review

## Abstract

Background/Objectives: Loneliness in adult cancer survivors is a frequent and disruptive psychosocial sequela with important consequences for overall health. Psychosocial interventions designed to address this issue are crucial. This systematic review and meta-analysis aimed to examine the efficacy of various psychosocial interventions for reducing loneliness in adult cancer survivors. Methods: A systematic literature search was conducted across PubMed, Web of Science, Scopus, Cochrane Library, PsycINFO, CINAHL Complete and Psicodoc for studies published up to October 2025. Methodological quality was assessed using PRISMA, RoB 2, and the PEDro Scale. The protocol was prospectively registered in PROSPERO (CRD420251174610). Results: Psychosocial interventions showed a significant and moderate effect on reducing loneliness (SMD = 0.46; 95% IC: 0.16 to 0.75). Heterogeneity was high (Q = 30.01; *p*-value < 0.001; I^2^ = 70%), and exploratory analyses suggested that differences in loneliness measurement instruments may contribute to this heterogeneity. Additional benefits were observed in anxiety, depression, well-being, and quality of life. Meaning-based and cognitive-behavioral approaches were found to be most effective. A large proportion of the studies showed a high risk of bias. Conclusions: Current evidence suggests that psychosocial interventions may reduce loneliness in adult cancer survivors. However, given the high risk of bias and heterogeneity, findings should be interpreted cautiously. Standardized tools and longitudinal designs remain essential.

## Simple Summary

Loneliness is a common and distressing challenge experienced by many adult cancer survivors, negatively impacting their emotional health and overall quality of life. This study reviewed and analyzed current scientific literature to determine whether psychological and social support programs are effective in reducing loneliness in this population. Our findings show that these support programs, particularly those focused on finding meaning and changing thought patterns, successfully reduce loneliness while also improving anxiety, depression, and general well-being. However, differences in how loneliness was measured across studies made comparisons challenging. These results emphasize the importance of incorporating psychological support into post-cancer care. Furthermore, this research highlights the need for researchers to adopt standardized measurement tools to better evaluate and improve emotional support programs for cancer survivors in the future.

## 1. Introduction

Population aging is one of the major health and social challenges of the 21st century. In high-income countries, more than 20% of the population is aged 65 years or older, reflecting a marked shift in demographic structure and a growing demand for complex social and healthcare services [1]. Life expectancy continues to rise worldwide, surpassing 80 years in many regions, with consistently higher female survival [2]. This demographic process increases the prevalence of chronic diseases, including cancer, which has been identified by the WHO as the second leading cause of death globally [3]. In 2022, 20 million new cancer diagnoses and 9.7 million cancer-related deaths were recorded worldwide, figures that are expected to continue increasing in the coming decades [4,5].

Beyond biological factors, cancer mortality and the lived experience of the disease are shaped by behavioral, social, and economic determinants. Smoking, alcohol consumption, obesity, and unhealthy diets account for nearly one-third of cancer-related deaths [3]. Added to these are age and socioeconomic inequalities, which influence survival and generate variability in access to healthcare even in high-income countries [6,7,8]. Moreover, cancer survivors present a wide range of physical and psychological symptoms—pain, fatigue, sleep disturbances, anxiety, and depression—that impair quality of life and hinder adaptation to the illness.

Among the psychosocial factors associated with poorer outcomes in this population, unwanted loneliness stands out and is defined as the subjective perception of lacking or having insufficient meaningful relationships [9]. Its association with premature mortality, cognitive decline, mental disorders, and cardiovascular diseases is well documented. During the COVID-19 pandemic, high levels of emotional distress, isolation, and loneliness were observed among older adults, highlighting the vulnerability of this group to social and health changes [10,11]. Gender differences and underlying biological mechanisms—chronic inflammation, immune dysfunction, and elevated markers of physiological stress—have also been described, contributing to its impact on health [12].

In oncology, this evidence is particularly compelling. Indeed, severe loneliness has been estimated to increase mortality risk by up to 67% among cancer survivors [13]. The transition to the survivorship phase, far from being free of challenges, can exacerbate loneliness due to physical changes, reduced perceived social support after active treatment, uncertainty about the future, and the reconfiguration of personal and social roles. Although psychosocial, behavioral, educational, digital, and peer-support interventions have been developed to tackle loneliness in this population, their effectiveness remains uncertain [14,15]. The available literature is heterogeneous, and findings from previous studies are inconsistent, making it difficult to establish robust recommendations for clinical practice and the design of specialized programs.

While previous efforts have been made to synthesize interventions for loneliness in cancer survivors—most notably the systematic review by McElfresh et al. [14]—an updated and methodologically expanded analysis is warranted for several key reasons. First, the search in McElfresh et al. concluded in May 2019 and identified only eight eligible studies. Since then, the landscape of psychosocial oncology has evolved significantly, particularly following the COVID-19 pandemic, which acted as a catalyst for novel digital, group, and nurse-led interventions. Second, beyond providing an updated synthesis up to October 2025, the current meta-analysis isolates loneliness as an independent primary survivorship outcome, expanding the evidence base to 11 eligible studies comprising 1011 participants, of which nine studies contributed 10 effect estimates to the quantitative synthesis, representing 666 unique participants. Third, earlier syntheses omitted advanced statistical modeling required to gauge real-world clinical applicability. By incorporating random-effects meta-analysis alongside 95% prediction intervals, rigorous risk-of-bias tools (RoB 2 and PEDro), publication bias diagnostics, and GRADE certainty assessments, this study provides a highly calibrated synthesis of effect sizes and their clinical predictability. Therefore, the aim of this study is to systematically synthesize and meta-analyze the effectiveness of psychosocial interventions in reducing loneliness among adult cancer survivors, offering operational insights for oncology nursing and interprofessional survivorship care.

## 2. Materials and Methods

This systematic review and meta-analysis were conducted following methodological recommendations of the Cochrane Collaboration. The reporting process adhered to the Preferred Reporting Items for Systematic Reviews and Meta-Analyses (PRISMA) [16]) (see Supplementary Materials S1 and S2) and followed the Enhancing the Quality and Transparency of Health Research (EQUATOR) network guidelines [17]. The study protocol was prospectively registered in the International Prospective Register of Systematic Reviews (PROSPERO; registration no. CRD420251174610).

### 2.1. Literature Search Strategy

A comprehensive search was performed across multiple electronic databases, including PubMed, Web of Science, Scopus, Cochrane Library, PsycINFO, CINAHL Complete, and Psicodoc, covering all publications available up to October 2025. The search strategy was structured around three main conceptual domains: cancer, loneliness, and intervention. Search terms related to cancer included “Cancer”, “Neoplasms”, “Neoplasm”, and “Tumor”; terms related to loneliness included “Loneliness”, “Isolation”, “Lonel*”, “Social isolation”, “Perceived social isolation”, and “Disconnectedness”; and the intervention block included “Intervention” and “Mindfulness”. Database-specific syntax and the number of records retrieved from each source are provided in the Supplementary File entitled “Explicit Search Entry” (Supplementary Material S3).

### 2.2. Eligibility Criteria

Inclusion criteria: All types of intervention studies that directly targeted loneliness among adult (≥18 years) cancer survivors, regardless of cancer type or cohort, were eligible for inclusion. Each intervention had to explicitly aim to address and reduce loneliness as a primary or central objective. Interventions could involve a single intervention group or be compared with a control group, an alternative intervention, a placebo, or a waitlist/sham condition. In addition, only studies that directly assessed loneliness using a validated measurement scale were included.

Exclusion criteria: Reviews or systematic reviews, intervention protocols, observational studies, single-group interventions combining multiple treatment components (i.e., not specifically focused on loneliness), single-case reports, conference abstracts, poster presentations, narrative reviews, and studies with unspecified or incomplete results.

### 2.3. Study Selection and Data Collection

In the first phase, two reviewers (ASG and JLSG) independently assessed the titles and abstracts of all retrieved records to identify potentially eligible studies. Full-text articles that appeared to meet the inclusion criteria were then obtained and evaluated in detail by both reviewers. Any discrepancies between their decisions were resolved by consulting a third reviewer (EJFR), who provided a final decision.

Data extraction was independently performed by ASG and JLSG in accordance with the guidelines of the Cochrane Handbook for Systematic Reviews of Interventions [18]. Any inconsistencies identified during this process were discussed and resolved through consensus among the research team. The extraction, finalized in November 2025, included information on study authorship, publication year, design, sample size, and main diagnosis.

### 2.4. Risk of Bias and Quality Assessments

The methodological quality and risk of bias of the randomized controlled trials included in this review were evaluated using the ROB2 tool [19] and the PEDro scale [20]. When discrepancies arose during the initial assessment, the original studies were re-examined, and consensus was reached through discussion among the reviewers.

### 2.5. Quantitative Data Synthesis and Analysis

The random effects model was selected because of the great variability in design, features of individuals and outcomes among the articles. The unbiased standardized mean difference (SMD) was employed as the effect size. The variability between studies (t2) was estimated using the restricted maximum likelihood procedure. The differences were calculated as pre-mean minus post-mean in each experimental group and subsequently intervention mean minus control mean. Therefore, positive values indicate an effect of the intervention. Heterogeneity was measured using Q-tests, I^2^ coefficients and prediction intervals proposed by Higgins et al. [21]. Baujat plot [22] was used to explore those studies that increase heterogeneity and are influential. Type of intervention, measure, cancer and PEDro’s quality scale were taken into account to explain heterogeneity by metapartition [23]. This technique is based on decomposing the sum of squares of the homogeneity test into the sum of squares between studies and the sum of squares within studies [24]. PEDro’s quality scale was considered a factor because the studies presented only two scores. Meta-regression has also been used to analyze the influence of treatment weeks, mean ages, and percentage of women on the variability of effect sizes. Publication bias was assessed using the contour-enhanced funnel plot, Egger’s test, Doi plot and LFK index procedures. The impact of effect size dependence was evaluated by calculating an integrated effect size from studies that provided more than one effect size. The 95% confidence intervals were calculated for effect sizes. The significance level was set at 5%. The analyses were carried out with the meta (version 8.2-1) and metasens (version 1.5-3) packages using R statistical software (version 4.5.2; R Foundation for Statistical Computing, Vienna, Austria).

## 3. Results

### 3.1. Study Selection

The initial search yielded a total of 10,288 records. After removing duplicates, 7603 records remained. Following title and abstract screening, 46 studies were considered potentially relevant, and their full reports were obtained and assessed. Out of these, 35 studies were excluded. Finally, 11 studies met the inclusion criteria, comprising a total of 1011 participants. Of these, nine studies were eligible for inclusion in the quantitative meta-analysis, contributing 10 effect estimates and representing 666 unique participants. The detailed screening process can be visualized in the PRISMA flow diagram (Figure 1).

### 3.2. Risk of Bias

ROB2 tool: Among the nine randomized controlled trials included, six studies (66%) were judged to have a high risk of bias, one (11%) had some concerns, and two studies (23%) were rated as having an overall low risk of bias (Figure 2A,B).

Among the five domains evaluated, the highest proportion of high risk of bias was observed in the domain related to the measurement of the outcome (D4), followed by the domain concerning missing outcome data (D3). In contrast, the domains assessing randomization process (D1), deviations from intended interventions (D2), and selection of the reported result (D5) predominantly showed low risk of bias.

The PEDro Scale: The methodological quality score ranged from 4 to 6 out of a maximum of 10 points. The mean methodological quality score of the studies was 4.7, indicating an overall moderate methodological quality. Most trials showed limitations related to blinding procedures and intention-to-treat analyses. Quality values for each clinical trial can be seen in Supplementary Material S4.

### 3.3. Characteristics of the Studies

The 11 selected studies [25,26,27,28,29,30,31,32,33,34,35] developed programs/interventions that directly addressed loneliness and evaluated this variable using a standardized instrument.

**Figure 2:**
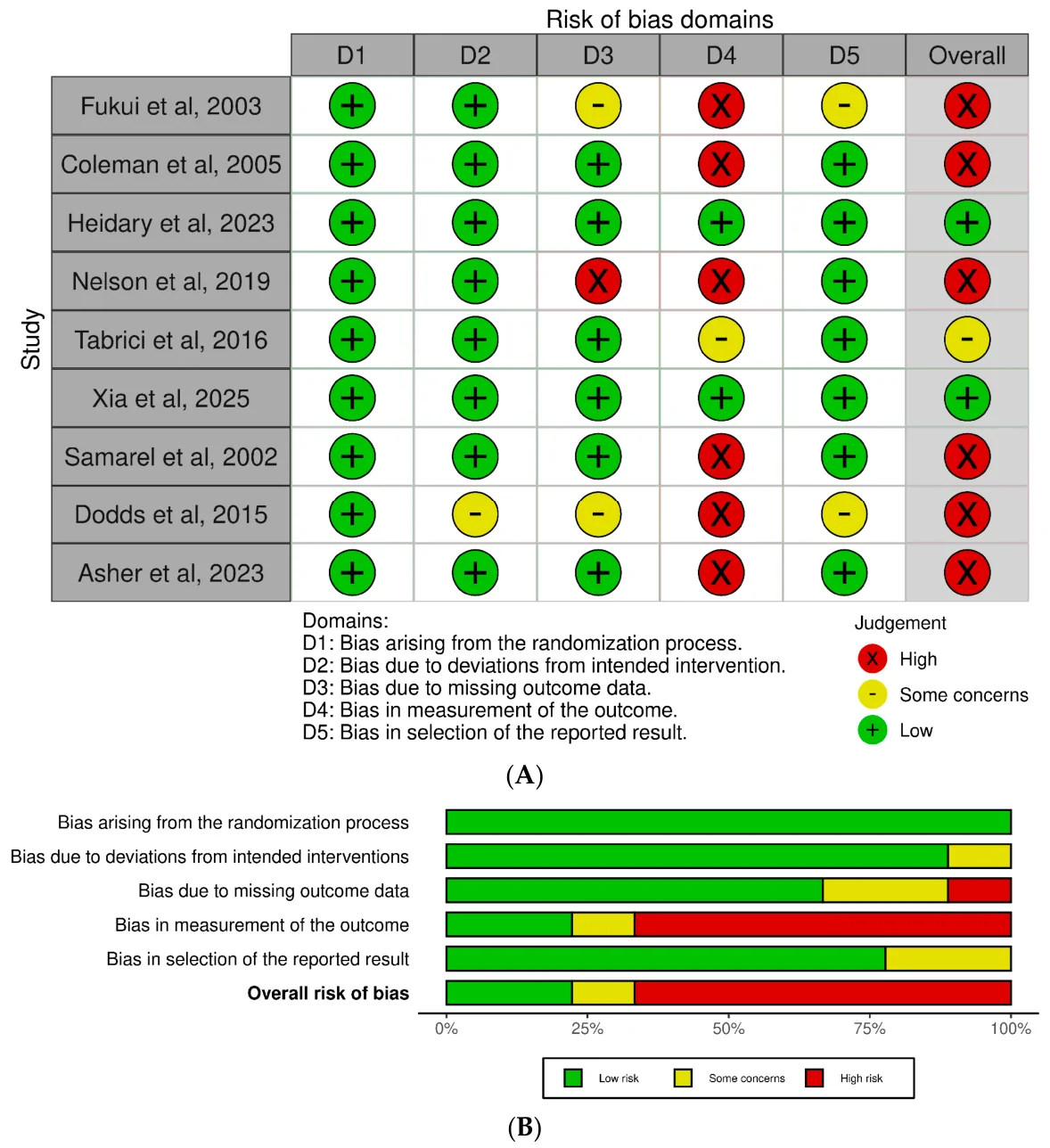
(**A**) Risk of Bias Assessment (1) [25,26,27,28,29,30,32,33,34]. (**B**) Risk of Bias Assessment (2).

However, most of them also considered and assessed other factors/variables, such as symptoms of stress or anxiety [26,27,28,30,33,34], symptoms of depression [28,30,31,33,34], quality of life or well-being [28,29,30,32,33,34] and social support [26,31].

Seven studies conducted follow-ups [25,28,29,31,33,34]. The effects of several interventions were generally sustained over time.

Detailed descriptions of each study and its primary outcome measures are provided in Table 1, while a full overview of study characteristics, intervention dosage, and secondary outcomes is available in Table S1 (Supplementary Material S10).

### 3.4. Sample Characteristics

The 11 studies included in the systematic review comprised a total of 1011 participants. Of these, nine studies contributed data to the quantitative meta-analysis, representing 666 unique participants across 10 effect estimates. Participants included breast cancer survivors [25,26,29,31,32,34,35], colorectal cancer survivors [30] or individuals with mixed cancer types [27,28,33] with a mean age of 56.29 years. Sample sizes ranged from 33 [33] to 197 participants [26], which reflects heterogeneity in the designs and scope of the interventions analyzed. The studies were carried out in several countries, i.e., Japan [25], the United States [26,28,31,32,33,34], Iran [27,29], China [30] or Egypt [35].

### 3.5. Characteristics of Interventions on Loneliness

Of the 11 studies included in this systematic review with meta-analysis, three evaluated meaning/existential-based interventions focusing primarily on the meaning of life, acceptance of reality and deep resilience [27,28,34].

Heidary et al. [27] implemented existential and meaning-centered psychotherapy with the experimental group (versus a waiting list control group) based on logotherapy. This intervention comprised ten 2-h sessions, one per week, and pursued the following objectives: introduction, clarification of creative, experiential and attitudinal values, focus on goals, fitting goals with values, setting new goals, planning for goal achievement, current status analysis and summary and critique. Nelson et al. [28] compared an experimental group, for whom they implemented meaning- and dignity-centered therapy, with a control group. The meaning-centered and dignity therapy consisted of five sessions (45 min) that took place over approximately seven weeks. All of them followed this pattern: reviewing the session topic, defining challenges associated with the topic, exploring “guided questions” surrounding the topic, assessing ways to reframe stressful emotions, thoughts, or behaviors, and identifying past coping skills or problem-solving related to the challenges. Asher et al. [34] implemented a program called GRACE with participants in the experimental group over eight weeks (one 60-min session per week), compared to a wait-list control group. GRACE incorporates both psycho-educational and experiential interventions. It was designed to provide a sense of awareness and control, develop ways of finding meaning and purpose, learn skills to enhance perspective management, strengthen connectivity, develop a sense of gratitude despite difficult circumstances, identify and utilize personal strengths and virtues, and to help crystallize one’s legacy as a means to living the most purposeful life possible.

Four studies implemented psychoeducational interventions designed to teach/train skills and provide information/resources for coping [25,30,33,35] (some of these interventions fall within specific types of therapy, such as cognitive behavioral therapy (CBT)) [30,33], or counseling [35]. Fukui et al. [25] compared a psychosocial group intervention (experimental group) with a waiting list control group. This intervention involved six 90-min sessions over six weeks, covering health education, coping skills training and stress management. Xia et al. [30] developed a standard nursing intervention (the same as those in the control group) along with cognitive behavioral stress management (CBSM) for 12 weeks. The CBSM-based intervention lasted for 90 min per week. Each session comprised 60 min of stress management and 30 min of relaxation training.

Dodds et al. [33] compared a Cognitively Based Compassion Training (CBCT) intervention, conducted over eight sessions (one per week, each lasting two hours), with a wait-list control group. The CBCT intervention included didactics, class discussion and guided meditation practice. The sessions focused on developing meditative concentration, developing a non-judgmental awareness of thoughts, internal bodily states and environmental circumstances (mindfulness), examining of the causes of suffering and practicing self-compassion, among other variables. In the study by El Kotait et al. [35] the experimental group participated in six counseling sessions. These included psychoeducation about the nature of breast cancer, transforming negative thoughts into positive thoughts to improve their perception of their bodies, training women to increase their self-esteem and self-confidence to help them to adjust to stress and improve their negative perception of their bodies, improving body image to increase their self-esteem, manual lymph drainage (MLD) to improve arm lymph edema, physical exercise, and nutritional advice. Each session continued for 45–60 min over 12 weeks. The control group received normal care.

Two studies implemented interventions centered on support and educational information. These interventions involved providing participants with educational information and offering them support and social assistance either by telephone or in person [26,32]. Coleman et al. [26] developed an intervention for their experimental group including educational information via email and telephone-based social support (as opposed to a control group that only received educational information via email). The intervention for the experimental group lasted 56 weeks and had four phases. Samarel et al. [32] compared an experimental group with two control groups. The experimental group received 13 months of combined individual telephone and in-person group support and education; control group 1 received 13 months of telephone-only individual support and education; and control group 2 received one-time mailed educational information.

Finally, two studies have been classified as supportive because they developed interventions emphasizing mutual support and emotional expression (supportive-expressive) [29,31]. The intervention developed by Tabrizi et al. [29] for their experimental group consisted of 12 weekly 90-min unstructured supportive-expressive discussion sessions. The leader during the sessions tried to facilitate the situation so that all participants could have a good opportunity to express fears and feelings, allowing participants to confront existential issues. The topics of the sessions consisted of different domains such as psychological information like stress of having cancer, fear of recurrence, explaining stress management, coping strategies for cancer, managing physical and mental activity, setting goals, being positive and having plans for the future and medical information about cancer. Cleary & Stanton [31] compared an internet-based psychosocial intervention in which participants received a manual with instructions and attended a three-hour workshop to enable them to use the specific functions of a website. This website included a diary (blog) to which participants could add photos and links to other websites of their choice. There was also a page titled “How you can help”, where participants could indicate to visitors the actions they could take. The authors mention that the distinctive feature of this intervention was that the participants were able to design a website based on their own preferences and needs. One week later, they received a follow-up phone call lasting no more than 15 min from a wait-list control group.

### 3.6. Qualitative Results

#### 3.6.1. Loneliness

The results for this variable were mixed. Four studies showed a significant improvement in levels of perceived loneliness in the post-intervention (participants perceived themselves as feeling less lonely) [25,27,30,35]. In the study of Fukui et al. [25], this improvement was also maintained at the 6-month follow-up. The other studies did not include follow-ups; two others obtained them at follow-up [31] at six months only, and Samarel et al. [32] at the end of phases II and III. However, four others did not obtain a significant difference in this variable after their intervention, either in the post-intervention or follow-up [26,28,29,33]. Only one study obtained a deterioration in the post-intervention and no changes in the follow-up [34].

Most studies [25,26,28,29,30,32,33,34] used the University of California Los Angeles Loneliness Scale (UCLA) to assess loneliness.

#### 3.6.2. Stress/Anxiety

Of the six studies that evaluated stress or anxiety symptoms [26,27,28,30,33,34], some also assessed related constructs such as cancer-related concerns [32] or fear of recurrent cancer [33]. However, only studies that directly measured anxiety symptoms were considered for the present conclusions. Of these, four reported a significant reduction in anxiety following the intervention [27,28,30,34]. In the study by Nelson et al. [28], these improvements were maintained at the 2-month follow-up. In contrast, the studies by Coleman et al. [26] and Dodds et al. [33] did not find significant differences at any assessment point.

#### 3.6.3. Depression

Five studies evaluated symptoms of depression [28,30,31,33,34]. Other studies also evaluated hopelessness [34] or demoralization [28], which can be considered related to depressive symptoms; however, only studies that evaluated depressive symptoms themselves were considered for these conclusions. Four of them showed an improvement in this variable after the intervention [28,30,33,34]. Of these, only one study observed that the improvement was maintained at follow-up [34], whereas another study reported a significant improvement emerging at follow-up but not immediately post-intervention [31].

#### 3.6.4. Quality of Life/Well-Being

Six studies assessed quality of life [29,33,34] or well-being [26,30,34]. Specifically, three studies examined spiritual well-being [28,30,34], and one study evaluated existential well-being [32].

All three studies measuring quality of life reported significant post-intervention improvements [29,33,34]; however, these effects were not sustained at follow-up in the study by Dodds et al. [33]. Similarly, all studies assessing spiritual well-being observed significant post-intervention improvements [28,30,34], with effects maintained at follow-up in two of them. In contrast, the study by Samarel et al. [32] did not report significant changes in existential well-being following the intervention.

#### 3.6.5. Social Support

With regard to the social support variable, one study reported a significant improvement at follow-up [31] and the other one [26] found no significant difference in social support in the post-intervention or follow-up assessments.

### 3.7. Quantitative Results

#### 3.7.1. Quantitative Results on Loneliness

The quantitative synthesis included 9 unique studies contributing 10 effect estimates and representing 666 unique participants. Ten effect estimates were included because Samarel et al. [32] contributed two comparisons sharing the same intervention group; therefore, these comparisons were treated as separate effect estimates but were not counted as separate studies or as additional unique participants. The overall SMD was 0.46 with a 95% confidence interval of 0.16 to 0.75 (Figure 3). Heterogeneity was high (Q = 30.01; *p*-value < 0.001; I^2^ = 70%). The 95% prediction interval (−0.39 to 1.30) was very wide and its values included both negative and positive. Available statistical and graphical assessments did not identify clear evidence of publication bias (Egger’s test = −0.12, *p* = 0.907; LFK index = 0.28). Complementary graphical inspections via the contour-enhanced funnel plot (Supplementary Material S5) and Doi plot (Supplementary Material S6) were similarly reassuring. However, given the modest number of included studies (N = 10), these diagnostics possess limited statistical power and should not be interpreted as definitive proof of the absence of publication bias. Additionally, the contour-enhanced funnel plot and Baujat plot (Supplementary Material S7) identified the effect size of Heidary’s study as an influential outlier.

If the Heidary study was eliminated, the effect size would be 0.35 (95%CI: 0.15 to 0.56). The Q test was 12.59 (*p*-value = 0.13) and I^2^ = 36,3%. Heidary’s study differs from other studies since it used the ELQ scale instead of the UCLA. The variability of the scores on this scale is small; it worked with mixed cancers, and it is also the only study in which there was a higher proportion of men than women (see Table 1).

Table 2 shows the results of the meta-partitioning and meta-regression based on the factors and quantitative variables. Only the instrument type factor explains the overall heterogeneity, generating three groups (Figure 4). Two of the groups relate to one study. The third group includes eight studies that use the UCLA questionnaire. The effect size of this group is 0.41 with a non-significant Q test (*p*-value = 0.305) and low heterogeneity explained by the studies (I^2^ = 15.8%).

No other factor or quantitative variable explains the overall heterogeneity. The forest plots for the factors are shown in the Supplementary Material S8A–C. The type of intervention shows similar effect sizes except for supported and educational information, which has a smaller effect size (SMD = 0.29). For meaning/existential-based intervention, the effect size is larger because Heidary’s study is the only group that shows significant heterogeneity.

#### 3.7.2. Sensitivity Analysis

Our study did not deviate from the protocol registered in PROSPERO. The effect size reported by Heidary et al. [27] represented an outlier. Although the effect size of this study did not significantly affect the overall pooled effect, it could have influenced the results of the subgroup analyses. Heterogeneity was high in the overall analysis, although exploratory analyses suggested that differences in the type of loneliness measurement instrument may partly account for this variability.

The results could also be affected by the dependence between the two effect estimates obtained from Samarel et al. [32], as both comparisons shared the same intervention group. Therefore, these two effect estimates were considered as originating from a single study and were not counted as independent studies or as additional unique participants. To assess the robustness of the findings, a sensitivity reanalysis was performed (Supplementary Material S9) including only one of the two effect estimates derived from Samarel et al. [32]. The resulting pooled effect size and confidence interval were similar to those obtained in the primary analysis (SMD = 0.48; 95% CI: 0.16 to 0.79). Heterogeneity estimates also remained broadly comparable (Q = 26.01; I^2^ = 69.2%). No clear evidence of publication bias was detected, although these findings should be interpreted cautiously given the limited number of studies.

### 3.8. Quality of Evidence

The certainty of evidence was assessed using the GRADE framework for the primary outcome included in the quantitative synthesis (Table 3). For loneliness, based on nine unique studies contributing 10 effect estimates and representing 666 unique participants, the certainty of evidence was rated as low. The evidence was downgraded due to serious risk of bias, as most randomized controlled trials presented methodological limitations, with six of nine trials classified as having a high overall risk of bias according to RoB 2. Certainty was further downgraded due to a serious inconsistency, given the substantial heterogeneity observed across studies (I^2^ = 70.3%), although sensitivity analyses suggested that part of this variability was influenced by an influential outlier study. Evidence was also downgraded for serious imprecision due to the limited number of participants and uncertainty regarding the magnitude and consistency of the effect in future clinical settings, reflected by the wide 95% prediction interval (−0.39 to 1.30). No downgrading was applied for indirectness, as the included studies directly addressed adult cancer survivors, psychosocial interventions, and validated loneliness outcomes. Additionally, available statistical and graphical assessments (Egger’s test, contour-enhanced funnel plot, Doi plot, and LFK index) did not identify clear evidence of publication bias, although these analyses possess limited statistical power and should be interpreted cautiously due to the small number of included studies. The pooled effect estimate showed a statistically significant reduction in loneliness following psychosocial interventions (SMD = 0.46; 95% CI: 0.16–0.75; 95% PI: −0.39 to 1.30).

## 4. Discussion

This systematic review and meta-analysis aimed to examine the efficacy of various psychosocial interventions (including existential, psychoeducational, cognitive-behavioral, supportive, and mixed approaches) for reducing loneliness levels in adult cancer survivors, one of the most frequent and emotionally disruptive psychosocial sequelae in this population [9]. The results of this study provide updated synthesized evidence on psychosocial interventions targeting loneliness in this population. While the pooled results show a statistically significant reduction in loneliness along with potential secondary benefits on anxiety, depression, and quality of life, these findings must be interpreted with caution due to the substantial methodological heterogeneity and high risk of bias across primary studies. These findings remain clinically promising, considering that loneliness is a growing problem in oncological survivorship and is associated with poorer quality of life and greater psychological symptomatology [13].

Notably, a significant proportion of the studies reported statistically significant improvements in loneliness, suggesting a promising strategy to mitigate this problem. These improvements were observed across different types of intervention, including existential and logotherapy programs [27,28,34], cognitive-behavioral approaches [30,33], and broad psychoeducational programs [25,35]. This is aligned with increasing evidence supporting the efficacy of psychological interventions in reducing loneliness in other clinical populations and reinforces the applicability of these approaches to oncological contexts.

The meta-analysis revealed a significant effect of moderate magnitude on loneliness (SMD = 0.46; 95% CI: 0.16 to 0.75), and available statistical and graphical assessments did not identify clear evidence of publication bias, although these diagnostics should be interpreted with caution given the small number of included studies. Crucially, while the pooled 95% confidence interval excludes zero—confirming a statistically significant average effect—the calculated 95% prediction interval ranged from −0.39 to 1.30. This distinction is clinically essential: whereas the confidence interval reflects the precision of the mean effect size across included studies, the prediction interval incorporates between-study heterogeneity to estimate the expected effect in a single new clinical setting. Because this interval spans across zero, the true effect of a psychosocial intervention in a future clinical setting could plausibly range from non-beneficial to substantially positive. Consequently, while current evidence supports a beneficial average effect, clinicians should recognize that intervention outcomes in real-world practice remain variable and uncertain. This effect is similar to or higher than that observed in previous meta-analyses focused on non-oncological populations (where SMDs are typically between 0.20 and 0.40) [36,37], suggesting that interventions designed for cancer survivors may be especially potent or better tailored to their specific emotional needs.

Our results specifically build upon and extend the findings of McElfresh et al. [14]. While their systematic review and meta-analysis included eight studies, our analysis incorporates 11. Specifically, our analysis shares seven core studies with the 2021 review but excludes one study because it was a meeting abstract and did not provide sufficient primary data for a full quality assessment and meta-analytical integration. Most importantly, we incorporate four recent high-quality trials published after the search period: Heidary et al. [27], Asher et al. [34], Hassan et al. [35], and Xia et al. [30]. This update is crucial, as it integrates evidence from the post-pandemic era. By including these recent studies, our findings provide a more robust and updated effect size (SMD = 0.46) compared to earlier syntheses. This comparison confirms that our study addresses a critical gap in the literature by integrating evidence from the post-pandemic era, during which loneliness in cancer survivors has been particularly prevalent.

The observed improvement in associated psychological variables (anxiety, depression, spiritual well-being, and quality of life) reinforces the idea that loneliness in survivors is not an isolated phenomenon, but a construct closely linked to emotional coping, perceived support, and post-treatment identity.

The majority of studies that evaluated anxiety [27,28,30,34] and depression [28,30,33,34] reported significant improvements, with the persistence of these effects observed in some cases during follow-up [34]. Interventions that evaluated spiritual or existential well-being [28,30,34] also reported statistically significant improvements, which is consistent with the literature highlighting the relevance of meaning and purpose components. Regarding quality of life [29,33,34], studies that measured it reported relevant improvements, although one study did not observe changes in existential well-being [32].

These findings are consistent with previous studies that have identified bidirectional associations between loneliness, mental health, and quality of life, reinforcing the idea that reducing loneliness can have benefits across different domains of emotional and social functioning [38,39,40].

The qualitative and quantitative analyses evaluated a range of therapeutic approaches. While meaning-based/existential interventions [27,28,34] and cognitive behavioral therapy (CBT)/psychoeducational programs [25,30,33,35] showed encouraging effect sizes in specific trials, these subgroup differences must be interpreted with substantial caution. Importantly, our meta-partitioning and meta-regression analyses demonstrated that intervention type did not significantly moderate overall treatment efficacy (*p* = 0.745). Furthermore, the apparently larger effect size observed in the meaning/existential subgroup was heavily driven by Heidary et al. [27], which was identified as an influential outlier. Consequently, comparisons between intervention modalities must be regarded as exploratory and hypothesis-generating rather than definitive evidence of the clinical superiority of any specific approach. On a conceptual level, however, these findings suggest that distinct mechanisms may contribute to mitigating loneliness: existential approaches (e.g., logotherapy or dignity therapy) may act by strengthening internal connection, narrative coherence, and purpose; CBT and psychoeducational strategies may operate by restructuring negative self-beliefs and improving coping skills; and supportive/educational approaches [26,29,31,32] may provide valuable structured social contact.

In summary, these findings reinforce the idea that loneliness is a modifiable phenomenon and that different approaches can contribute to reducing it, especially when they integrate emotional, cognitive, and existential components. Furthermore, on a hypothetical level, the core therapeutic mechanisms underlying these effective interventions—such as existential reflection, cognitive restructuring, and structured social connectedness—may extend beyond oncology. Individuals facing other chronic, life-threatening, or disabling conditions (e.g., neurodegenerative, cardiovascular, or autoimmune diseases) often experience comparable psychosocial challenges, including existential distress, altered personal identity, and social isolation. Thus, it is plausible that similar psychosocial interventions could be adapted to effectively mitigate loneliness across broader chronic illness populations, a hypothesis that warrants further empirical investigation.

### 4.1. Study Limitations

Despite the positive results and methodological strengths, several challenges limit the generalization of these findings. Beyond measurement tools, substantial clinical and methodological heterogeneity across primary trials remains a central limitation. As detailed in Table S1, the included studies exhibited marked variations in intervention content, dosage, duration, delivery modality (e.g., individual, group, or digital), control conditions (e.g., waitlist, usual care, or active education), cancer site, participant age, and follow-up timelines. The lack of standardization in intervention protocols hinders direct comparison between results and quantitative synthesis.

Although the overall pooled effect remained consistent, heterogeneity was substantial across included studies. In our exploratory subgroup and meta-regression analyses, the measurement instrument emerged as the strongest identified source of heterogeneity. However, this finding must be interpreted cautiously: while eight studies utilized the UCLA, the remaining two instrument subgroups comprised only a single study each. Given the modest number of included trials (N = 10), moderator analyses are inherently vulnerable to statistical instability, and instrument type should be considered a candidate explanatory factor rather than a conclusive, all-encompassing explanation for the observed heterogeneity. The broader adoption of standardized and validated tools, such as the UCLA, will be crucial for future meta-analytic syntheses.

Furthermore, the small number of included studies limits the statistical power and reliability not only of subgroup analyses but also of publication-bias diagnostics (such as Egger’s test and graphical funnel plots), which should not be interpreted as definitive proof of the absence of reporting bias.

The predominance of women with breast cancer and the scarcity of studies in other cancer types and in men may also limit generalization. Likewise, the fact that only a minority of studies conducted follow-up assessments restricts the capacity to determine the long-term durability of the effects and identify the psychological mechanisms involved in the persistence of benefits. Furthermore, studies that conducted interventions with this population focused on evaluating social support, rather than loneliness, and were therefore excluded. Finally, not all studies evaluated relevant secondary variables like anxiety or depression, despite their clear clinical relevance.

### 4.2. Clinical Implications

The results of this meta-analysis offer relevant insights for clinical practice in oncology, emphasizing that loneliness is a modifiable survivorship outcome closely linked to psychological distress, depression, anxiety, and reduced quality of life. Given their longitudinal presence across active treatment, symptom management, and long-term survivorship, oncology nurses and interprofessional healthcare teams are uniquely positioned to translate these findings into routine clinical care.

Rather than viewing loneliness management solely as a specialized secondary referral, practice models should integrate routine assessment of loneliness and social connectedness where clinically appropriate during survivorship care visits. Where formal screening programs are implemented, they must be strictly supported by established interprofessional referral pathways and adequate clinical resources to ensure that identified distress is met with actionable care. Oncology nurses can lead early risk identification, deliver brief nurse-led psychoeducational and coping-skills interventions, and incorporate social well-being assessments into survivorship care planning. When severe psychological distress, depression, or complex existential burden is identified, these clear interprofessional pathways facilitate timely escalation to specialized psycho-oncology or mental health services.

Importantly, current evidence does not justify prescribing any single intervention type as a universal standard of care. Given the predominance of breast cancer survivors in the available literature, variations in intervention dosage and protocols, and the wide prediction interval observed in our meta-analysis, clinical recommendations must remain cautious and tailored. Interprofessional care should move toward personalized oncology care, matching individual patient preferences, cancer type, and survivorship stage with structured psychosocial resources rather than assuming a one-size-fits-all approach.

## 5. Conclusions

Overall, current evidence suggests that psychosocial interventions may produce a moderate average reduction in loneliness among adult cancer survivors, accompanied by potential secondary benefits in mental health and overall well-being. However, confidence in this pooled estimate remains limited by substantial heterogeneity, serious risk of bias, imprecision, and uncertainty regarding effects in future clinical settings, as reflected by a prediction interval crossing zero. Given these limitations, current findings support the consideration and individualized implementation of these programs within survivorship care, rather than a universal standard of care, while highlighting the need for rigorous longitudinal studies to examine long-term intervention durability and optimal treatment matching.

## Figures and Tables

**Figure 1:**
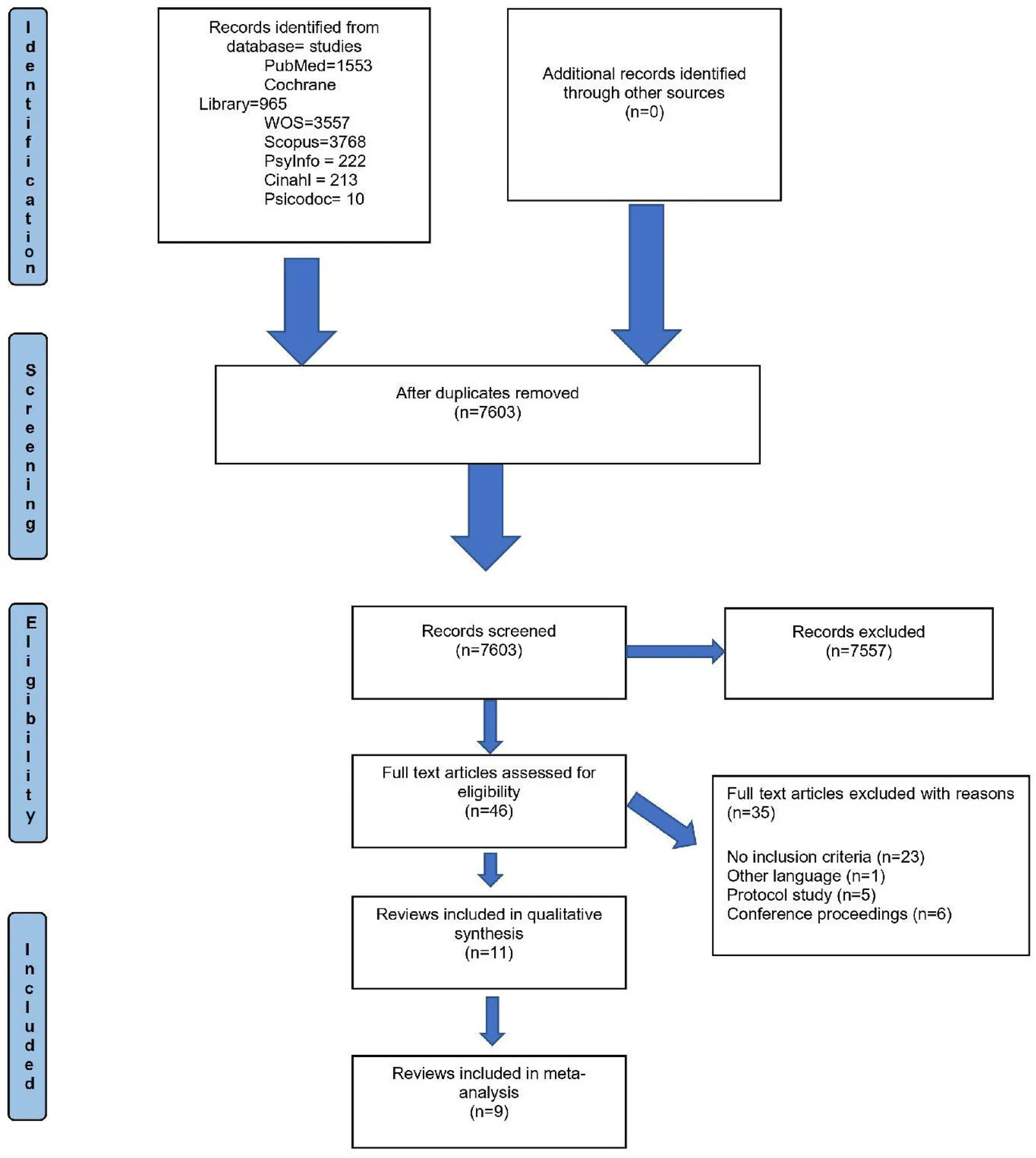
Flow diagram.

**Figure 3:**
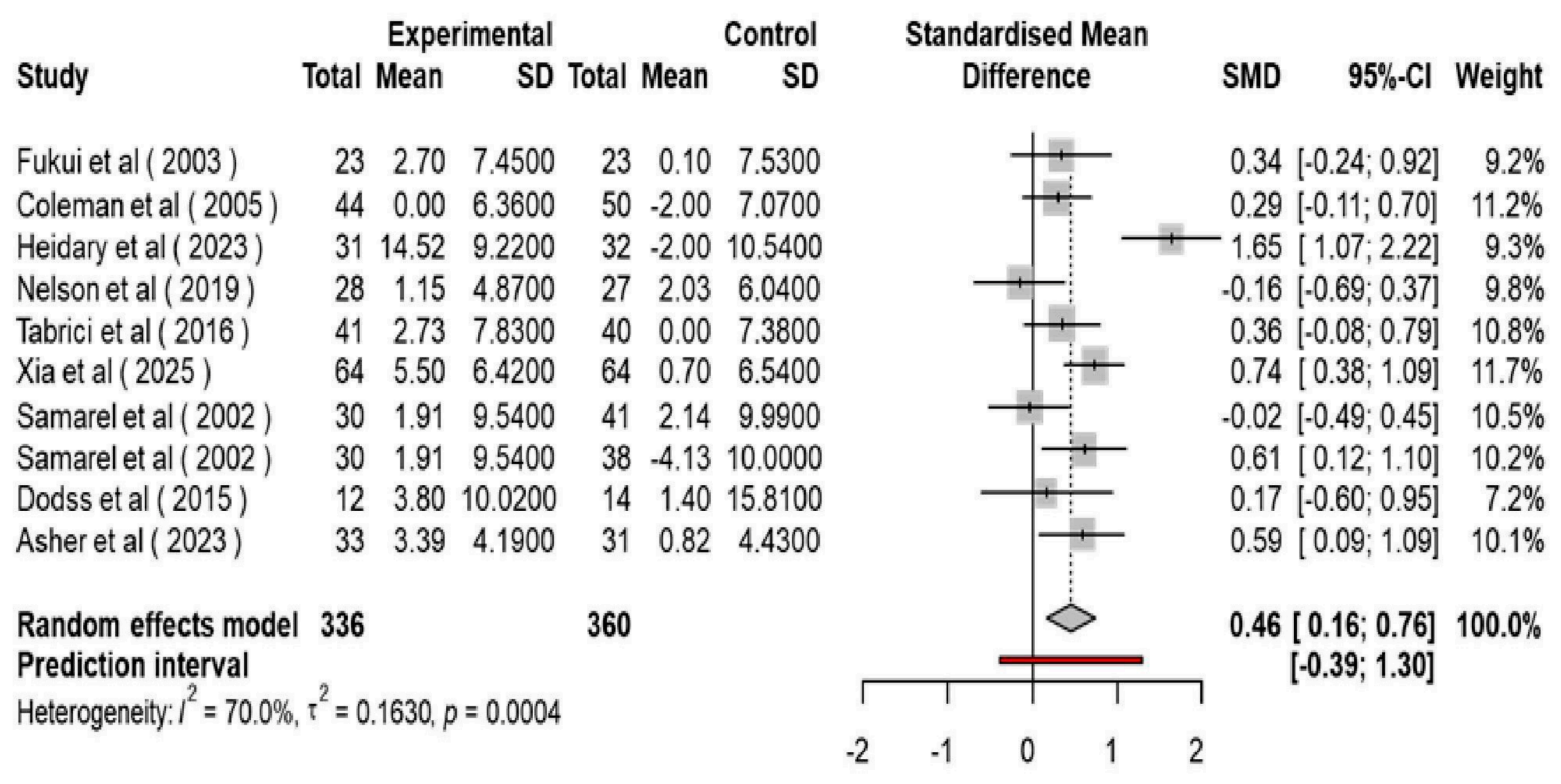
The forest plot of the loneliness scale. Standardized mean difference (SMD) and standard error (SE) [25,26,27,28,29,30,32,33,34].

**Figure 4:**
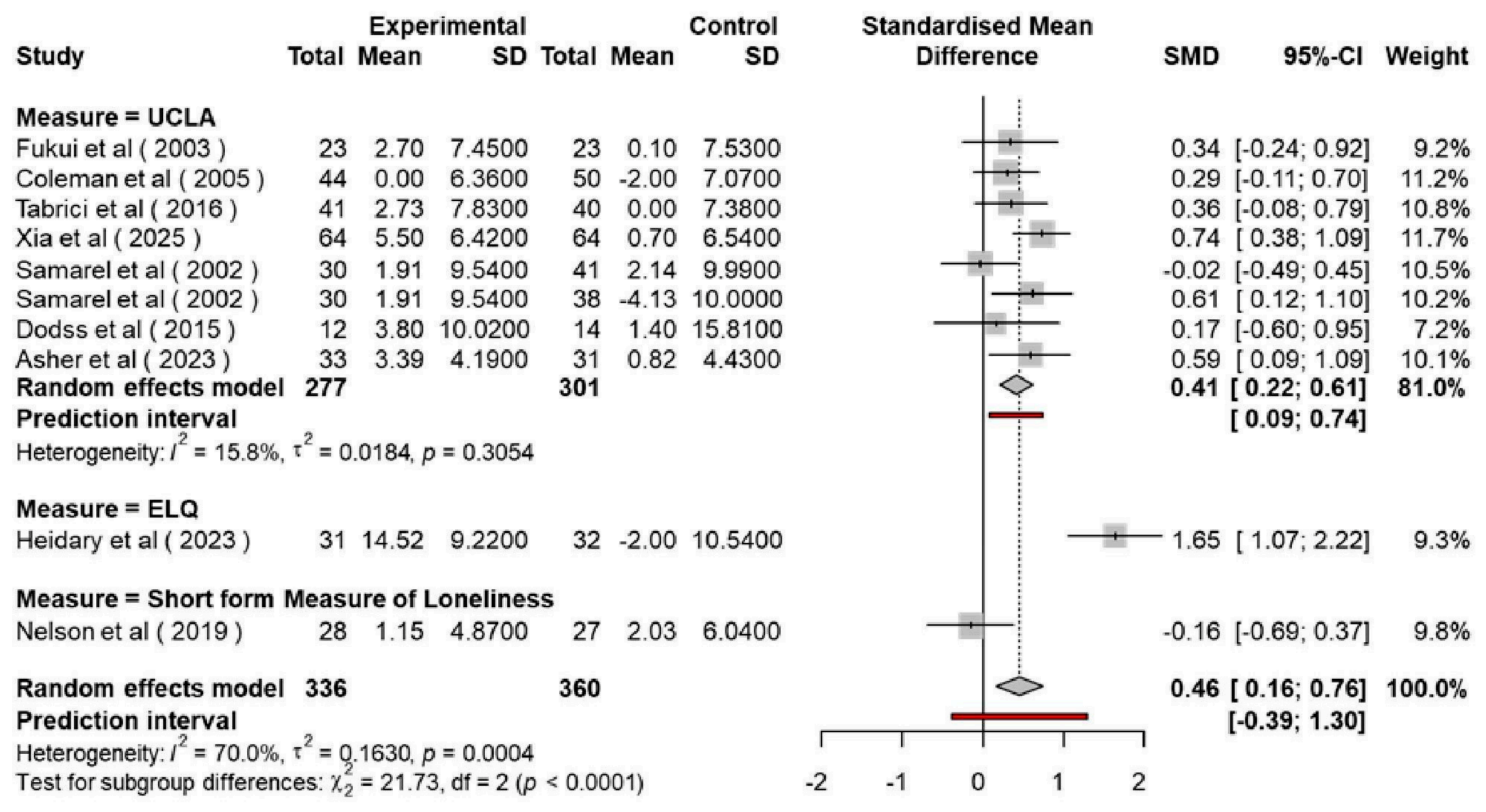
The forest plot for partitioning by measurement instrument. Standardized mean difference (SMD) and standard error (SE) [25,26,27,28,29,30,32,33,34].

**Table 1:** Main characteristics and primary loneliness outcomes of included studies.

Study	Country	Design	N	Cancer Type	Intervention Classification	Loneliness Measure	Main Loneliness Findings
Fukui et al. [25]	Japan	RCT	50	Breast Cancer	Psychoeducational	UCLA	IG ↑ UCLA (*p* < 0.05) vs. CG at Post; maintained at 6-mo FU.
Coleman et al. [26]	United States	Experimental	197	Breast Cancer	Support & Educational	UCLA	IG ↔ UCLA vs. CG across all phases.
Heidary et al. [27]	Iran	RCT	63	Mixed Cancer	Meaning/Existential	ELQ	IG ↑ ELQ (*p* < 0.001) vs. CG at Post.
Nelson et al. [28]	United States	Pilot RCT	59	Mixed Cancer	Meaning/Existential	UCLA	Post & FU: IG ↔ UCLA (*p* > 0.05) vs. CG.
Tabrizi et al. [29]	Iran	RCT	81	Breast Cancer	Support	UCLA	IG ↑ UCLA (*p* < 0.001) vs. CG at Post; maintained at 2-mo FU.
Xia [30]	China	RCT	128	Colorectal Cancer	Psychoeducational	UCLA	IG ↑ UCLA (*p* < 0.05) vs. CG at Post.
Cleary & Stanton [31]	United States	RCT	88	Breast Cancer	Support	UCLA	IG ↑ UCLA (*p* < 0.05) vs. CG at 6-mo FU.
Samarel et al. [32]	United States	RCT	125	Breast Cancer	Support & Educational	UCLA	IG & CG1 ↑ UCLA (*p* < 0.05) at end of phases II/III vs. CG2.
Dodds et al. [33]	United States	RCT	33	Mixed Cancer	Psychoeducational	UCLA	Post & FU: IG ↔ UCLA (*p* > 0.05) vs. CG.
Asher et al. [34]	United States	RCT	71	Breast Cancer	Meaning/Existential	UCLA	Post: IG ↓ UCLA (*p* < 0.001) vs. CG; FU: IG ↔ UCLA (*p* = 0.108).
Hassan El-Kotait et al. [35]	Egypt	RCT	116	Breast Cancer	Psychoeducational	CLS	IG ↑ CLS (*p* < 0.001) vs. CG at Post.

Note: RCT = Randomized Controlled Trial; UCLA = University of California Los Angeles Loneliness Scale; ELQ = Existential Loneliness Questionnaire; CLS = Cancer Loneliness Scale; IG = Intervention Group; CG = Control Group; FU = Follow-up; ↑ = Improvement; ↓ = Deterioration; ↔ = No change.

**Table 2:** Between-class fit statistics (QB) and *p*-values for moderate variables.

**Meta-Partition**	**Q_B_**	***p*** **-Value**
Type of measure	21.73	<0.001
Type of intervention	1.23	0.745
Type of cancer	3.50	0.174
PEDro Scale of quality	1.25	0.274
**Meta-Regression**	**Q_B_**	***p*** **-Value**
Weeks of Intervention	0.379	0.534
% lost	1.405	0.237
% of women in control group	1.363	0.243
% of women in intervention group	1.715	0.190
Mean age in control group (years)	1.180	0.277
Mean age in intervention group (years)	0.593	0.441

**Table 3:** GRADE evidence profile for psychosocial interventions targeting loneliness among adult cancer survivors.

Outcome, Studies, and Sample Size	Risk of Bias ^a^	Inconsistency ^b^	Indirectness ^c^	Imprecision ^d^	Publication Bias ^e^	Effect Estima-Te (95% CI) ^f^	Quality of Evidence
Loneliness							
9 studies (*n* = 666)	Serious	Serious (*I*^2^ = 70%)	Not serious	Serious	Not serious	SMD = 0.46 (95% CI: 0.16 to 0.75)	Low

GRADE: Grading of Recommendations Assessment, Development, and Evaluation. ^a^ No: Most information is derived from studies with a low risk of bias. Serious: Important methodological limitations exist that may reduce confidence in the estimate of effect. Very serious: Limitations are sufficient to substantially reduce confidence in the estimated effect. ^b^ Serious: I^2^ > 40%; very serious: I^2^ > 80%. ^c^ No indirectness of evidence was identified because included studies directly addressed adult cancer survivors, psychosocial interventions, and validated loneliness outcomes. ^d^ Serious: Limited number of participants and uncertainty regarding the magnitude and consistency of effects in future clinical settings, reflected by the wide 95% prediction interval (−0.39 to 1.30). ^e^ Not serious: Statistical and graphical assessments (Egger’s test, contour-enhanced funnel plot, Doi plot, and LFK index) did not identify clear evidence of publication bias; however, these analyses are limited by the small number of studies. ^f^ SMD = Standardized Mean Difference.

## Data Availability

The data that support the findings of this study are available in the Supplementary Material of this article.

## Supplementary Materials

The following supporting information can be downloaded at https://www.mdpi.com/article/10.3390/cancers18193101/s1, Supplementary Material S1: PRISMA 2020 Statement and Checklist; Supplementary Material S2: PRISMA 2020 Abstract Checklist; Supplementary Material S3: Explicit Search Entry; Supplementary Material S4: Methodological score of randomized clinical trials; Supplementary Material S5: The contour-enhanced funnel plot; Supplementary Material S6: Doi plot; Supplementary Material S7: Contour-enhanced funnel plot; Supplementary Material S8A: The forest plot for partitioning by type of intervention; Supplementary Material S8B: The forest plot for partitioning by type of cancer; Supplementary Material S8C: The forest plot for partitioning by PEDro scale of quality; Supplementary Material S9: Reanalysis forest plot; Supplementary Material S10: Table S1. Detailed characteristics.

## Abbreviations

The following abbreviations are used in this manuscript:

CES-D	Center for Epidemiologic Studies Depression Scale
CES-D-10	Brief Center for Epidemiologic Studies Depression Questionnaire
CG	Control Group
CLS	Cancer Loneliness Scale
DAS	Templer Death Anxiety Scale
ELQ	Existential Loneliness Questionnaire
EORTC QLQ-C30	European Organization for Research and Treatment of Cancer
EWBS	Existential Well-being Questionnaire
FACIT	Spiritual Well-Being Scale
FACIT-Sp	Functional Assessment of Chronic Illness Therapy-Spiritual Well-being Scale
FACT-G	Functional Assessment of Cancer Therapy General
IG	Intervention Group
NR	Not Reported
PHQ-9	Patient Health Questionnaire-9
PROMIS	Patient-Reported Outcomes Measurement Information System (PROMIS) Anxiety
PSDS	Physical Symptom Distress Scale
PSS-4	Perceived Stress Scale-4
RCT	Randomized Controlled Trial
SAS	Zung Self-Rating Anxiety Scale
SDS	Zung Self-Rating Depression Scale
SF-12	Medical Outcomes Study Short Form 12-Item Health Survey
UCLA	University of California Los Angeles Loneliness Scale
VAS-R	Visual Analogue Scale for Relationship Change with Significant Others
WHO	World Health Organization
