# Supplementary material for: Effectiveness of Interventions for Reducing Loneliness Among Adult Cancer Survivors: A Systematic Review and Meta-Analysis
Source: Cancers (Basel). 2026 Sep 24;18(19):3101. doi: 10.3390/cancers18193101 (PMC13664572; doi:10.3390/cancers18193101)
Supplement: Supplementary file 1 [file cancers-18-03101-s001.zip › Supplementary Material S10. Table S1. Detailed characteristics.pdf]

Table S1. Detailed characteristics, dosage, follow-up, and secondary outcomes of included studies.

Study	Country	Study Design	N	Type of cancer	Groups (n)	Age (years)	Type of Interven-tion	Classification of the type of intervention	Intervention volume			Follow-up	Assessments					Main Outcomes
									Weeks	Frequency (Sessions/ Week)	Session Duration (Minutes)		Loneliness	Stress/ Anxiety	Depression	Quality of life/ Well-being	Social support	
Fukui et al, 2003	Japan	RCT	50	Breast Cancer	Intervention group (25)	52.6	Psychosocial group intervention	Psychoeducational Interventions	6	1 session / week	90	6 months	UCLA	NR	NR	NR	NR	IG ↑ UCLA (p<0.05) in comparison with GC at Post and the effect was maintained at Follow-up (6 months).
					Control group (25)	54.3	Wait-list control group											
Coleman et al, 2005	United States	Experimental Study	197	Breast Cancer	Intervention group (54)	53,5	Educational information by e-mail and social support by telephone	Support and educational information interventions	56	Phase I: 1 session / week<br>Phase II: 1 session / week<br>Phase III: 2 session / month<br>Phase IV: 1 session / month	20-40	No	UCLA	PSDS	NR	NR	NR	IG ↔ UCLA, PSDS in comparison with GC in any of the phases.
					Control group (52)	52,8	Educational information by e-mail											
Heidary et al, 2023	Iran	RCT	63	Mixed Cancer	Intervention group (31)	53.93	Existential and meaning centered psychotherapy	Meaning/Existential-based interventions	10	1 session / week	120	No	ELQ	DAS	NR	NR	NR	IG ↑ ELQ (p<0.001), DAS (p<0.001) in comparison with GC at Post

					Control Group (32)	52.56	Wait-list control group										
Nelson et al, 2018	United States	Pilot RCT	59	Mixed Cancer	Intervention group (31)	76	Meaning-centered and dignity therapy	Meaning/Existential-based interventions	7	1 session / week	45	2 months	UCLA	HADS-A	HADS-D	FACIT	NR	Post: IG ↔ UCLA ($p>0.05$), HADS-A ($p>0.05$), FACIT ($p>0.05$), ↑ HADS-D ($p<0.05$) in comparison with GC.<br><br>Follow-up: ↔ UCLA ($p>0.05$), HADS-A ($p>0.05$), HADS-D ($p>0.05$), ↑ FACIT ($p<0.05$) in comparison with GC.
					Control Group (28)		Enhanced Social Work Control (ESWC)										
Tabrizi et al, 2016	Iran	RCT	81	Breast Cancer	Intervention group (41)	47.9	Supportive-expressive group therapy	Support interventions	12	1 session / week	90	2 months	UCLA	NR	NR	EORTC QLQ-C30	NR	IG ↑ UCLA ($p<0.001$), EORTC QLQ-C30 ($p<0.05$) in comparison with GC at Post and the effect was maintained at follow-up (2 months)
					Control Group (40)		Normal Care (NC)										
Xia, 2025	China	RCT	128	Colorectal cancer	Intervention group (64)	62.9	Cognitive-behavioral therapy	Psychoeducational Interventions	12	1 session / week	90	No	UCLA	SAS	SDS	FACIT	NR	IG ↑ UCLA ($p<0.05$), SAS ($p<0.05$), SDS ($p<0.05$), FACIT ($p<0.05$) in comparison
					Control Group (64)	62	Normal Care (NC)										

																with GC at Post.
Cleary & Stanton, 2014	United States	RCT	88	Breast Cancer	Intervention group (46)	55	Internet-based psychosocial intervention_	Support interventions	One 3-hour workshop session and a follow-up phone call one week later lasting no more than 15 minutes.	1 and 6 months	UCLA	NR	CES-D	NR	Sources of Social Support Scale	IG ↑ UCLA (p<0.05), CES-D (p<0.05), Sources of Social Support Scale (p<0.05) in comparison with GC at 6 months.
					Control Group (42)	56	Wait-list control group									
Samarel et al, 2002	United States	RCT	125	Breast Cancer	Intervention group (34)	53.8	In person group support and education and individual telephone	Support and educational information interventions	Total 13 months. Phase I: 8-10 weeks, during which weekly calls were made for 10-20 minutes. 8 to 10 sessions.<br><br>Phase II: 8 weeks. Face-to-face group sessions. 8 sessions of 2 hours.<br><br>Phase III: 8 months. Every two months, a 10-20 minute phone call was made. Total 11 sessions.	No	UCLA	NR	NR	EWBS	NR	IG and CG1 ↑ UCLA (p<0.05) at the end of phases II and III in comparison with CG2. And ↔ EWBS (p>0.05) between the three groups.
					Control group 1 (48)		Only individual support and education by telephone		Total 13 months. Phase I: 8-10 weeks. Weekly phone support, 10-20 minutes per call. 8 to 10 sessions.<br><br>Phase II: 8 weeks. Weekly telephone support of 10 to 20 minutes per call. 8 sessions.<br><br>Phase III: 8 months. Every two months, a 10- to 20-minute phone call was made. 11 sessions.							
					Control group 2 (43)		One-time mailed educational information		A single intervention consisting of sending an email with educational information at the beginning of the 13 months.							

Dodds et al 2015	United States	RCT	33	Mixed Cancer	Intervention group (16)	54.7	Cognitively-Based Compassion Training (CBCT)	Psychoeducational Interventions	8	1 session / week	120	1 month	UCLA	PSS-4	CES-D-10	SF-12	NR	Post: IG ↔ UCLA ($p>0.05$), PSS-4 ($p>0.05$), ↑ CES-D-10 ($p<0.01$), SF-12 ($p<0.01$) in comparison with GC.<br><br>Follow-up: IG ↔ UCLA ($p>0.05$), CES-D-10 ($p>0.05$), ↑ PSS-4 ($p<0.05$), SF-12 ($p<0.05$) in comparison with GC.
					Control Group (17)	55.8	Wait-list control group											
Asher et al, 2023	United States	RCT	71	Breast Cancer	Intervention group (37)	63.5	Psycho-educational and experiential program (GRACE)	Meaning/Existential-based interventions	8	1 session / week	60	1 month	UCLA	PROMIS	PHQ-9	FACT-G and FACIT-Sp	NR	Post: IG ↓ UCLA ($p<0.001$), ↑ PROMIS ($p<0.05$), PHQ-9 ($p<0.001$), FACT-G ($p<0.05$) and FACT-Sp ($p<0.05$) in comparison with CG.<br><br>Follow-up: IG ↔ UCLA ($p=0.108$), PROMIS ($p>0.05$), ↑ FACT-G ($p<0.05$) and FACT-Sp ($p<0.05$) and PHQ-9 ($P<0.05$) in
					Control Group (34)	59.5	Wait-list control group											

																		comparison with CG.
Hassan El-Kotait et al, 2024	Egypt	RCT	116	Breast Cancer	Intervention group (58)	45.67	Counselling	Psychoeducational Interventions	12	1 session/2 weeks (6 sessions in total)	45-60	No	CLS	NR	NR	NR	NR	IG ↑ CLS (p<0.001) in comparison with CG .
					Control Group (58)	48.1	Normal care (NC)											

RCT = Randomized Controlled Trial; UCLA=University of California Los Angeles Loneliness Scale; ELQ = Existential Loneliness Questionnaire; CLS = Cancer Loneliness Scale; PSS-4 = Perceived Stress Scale - 4; PROMIS = Patient Reported Outcomes Measurement Information System (PROMIS) Anxiety; CES-D = Center for Epidemiologic Studies Depression Scale; CES-D-10 = Brief Center for Epidemiologic studies - Depression questionnaire; PHQ-9 = Patient Health Questionnaire - 9; EWBS = Existential Well-being Questionnaire; SF-12 = Medical Outcomes Study Short Form 12 - Item Health Survey; FACT-G = Functional Assessment of Cancer Therapy General; FACIT-Sp = Functional Assessment o chronic illness Therapy-Spiritual Well-being Scale; IG: Intervention Group; CG: Control Group; ↑: Improvement; ↓: Deterioration; ↔: No changes. NR: Not Reported;; PSDS=Physical Symptom Distress Scale; VAS-R= Visual Analogue Scale for Relationship Change with Significant Others; DAS=Templer Death Anxiety Scale; FACIT=Spiritual Well-Being Scale; EORTC QLQ-C30=European Organization for Research and Treatment of Cancer; SAS=Zung Self-Rating Anxiety Scale; SDS=Zung Self-Rating Depression Scale.
