# Supplementary material for: Effectiveness of Interventions for Reducing Loneliness Among Adult Cancer Survivors: A Systematic Review and Meta-Analysis
Source: Cancers (Basel). 2026 Sep 24;18(19):3101. doi: 10.3390/cancers18193101 (PMC13664572; doi:10.3390/cancers18193101)
Supplement: Supplementary file 1 [file cancers-18-03101-s001.zip › Supplementary Material S3. Explicit Search Entry.pdf]

Supplementary Material S3: Explicit Search Entry (21/10/2025)

PubMed Search Formula: 1553

("Cancer" OR "Neoplasms" OR "Neoplasm" OR "Tumor") AND ("Loneliness" OR "Isolation" OR "Lonel*" OR "Social isolation" OR "Perceived social isolation" OR "Disconnectedness") AND ("Intervention" OR "Mindfulness")

Cochrane Library Search Formula: 965

("Cancer" OR "Neoplasms" OR "Neoplasm" OR "Tumor") AND ("Loneliness" OR "Isolation" OR "Lonel*" OR "Social isolation" OR "Perceived social isolation" OR "Disconnectedness") AND ("Intervention" OR "Mindfulness") in Title Abstract Keyword

SCOPUS Search Formula: 3768

TITLE-ABS-KEY ("Cancer" OR "Neoplasms" OR "Neoplasm" OR "Tumor") AND ("Loneliness" OR "Isolation" OR "Lonel*" OR "Social isolation" OR "Perceived social isolation" OR "Disconnectedness") AND ("Intervention" OR "Mindfulness")

WOS Search Formula: 3557

("Cancer" OR "Neoplasms" OR "Neoplasm" OR "Tumor") AND ("Loneliness" OR "Isolation" OR "Lonel*" OR "Social isolation" OR "Perceived social isolation" OR "Disconnectedness") AND ("Intervention" OR "Mindfulness") en Título Resumen Palabra clave (All Fields)

Psicodoc: 10

("Cancer" OR "Neoplasms" OR "Neoplasm" OR "Tumor") AND ("Loneliness" OR "Isolation" OR "Lonel*" OR "Social isolation" OR "Perceived social isolation" OR "Disconnectedness") AND ("Intervention" OR "Mindfulness") (All Fields)

Cinahl: 213

("Cancer" OR "Neoplasms" OR "Neoplasm" OR "Tumor") AND ("Loneliness" OR "Isolation" OR "Lonel*" OR "Social isolation" OR "Perceived social isolation" OR "Disconnectedness") AND ("Intervention" OR "Mindfulness") (All Fields)

PsyINFO: 222

("Cancer" OR "Neoplasms" OR "Neoplasm" OR "Tumor") AND ("Loneliness" OR "Isolation" OR "Lonel*" OR "Social isolation" OR "Perceived social isolation" OR "Disconnectedness") AND ("Intervention" OR "Mindfulness") (All Fields)
