# Supplementary material for: Effectiveness of Interventions for Reducing Loneliness Among Adult Cancer Survivors: A Systematic Review and Meta-Analysis
Source: Cancers (Basel). 2026 Sep 24;18(19):3101. doi: 10.3390/cancers18193101 (PMC13664572; doi:10.3390/cancers18193101)
Supplement: Supplementary file 1 [file cancers-18-03101-s001.zip › Supplementary Material S4. Methodological score of randomized clinical trials.pdf]

Supplementary Material S4. Methodological score of randomized clinical trials using the Physiotherapy Evidence Database (PEDro) scale.

Study	1	2	3	4	5	6	7	8	9	10	11	Total
Fukui et al, 2003	1	1	0	1	0	0	0	1	0	1	1	5/10
Coleman et al, 2005	1	1	0	1	0	0	0	0	0	1	1	4/10
Heidary et al, 2023	1	1	0	1	0	0	0	1	0	1	1	5/10
Nelson et al, 2019	1	1	0	1	0	0	1	0	0	1	1	5/10
Tabrici et al, 2016	1	1	0	1	0	0	0	1	0	1	1	5/10
Xia et al, 2025	1	1	0	1	0	0	0	1	0	1	1	5/10
Cleary et al, 2014	1	1	0	1	0	0	0	0	0	1	1	4/10
Samarel et al, 2002	1	1	0	1	0	0	0	0	0	1	1	4/10
Dodds et al, 2015	1	1	0	1	0	0	0	0	0	1	1	4/10
Asher et al, 2023	1	1	0	1	0	0	0	1	0	1	1	5/10
El Kotait et al, 2024	1	1	1	1	0	0	0	1	0	1	1	6/10

1: eligibility criteria; 2: random allocation of participants; 3: concealed allocation; 4: similarity between groups at baseline; 5: participant blinding; 6: therapist blinding; 7: assessor blinding; 8: dropout rate less than 15%; 9: intention-to-treat analysis; 10: between-group statistical comparisons; 11: point measures and variability data.
